# Identification of potential serum biomarkers for congenital heart disease children with pulmonary arterial hypertension by metabonomics

**DOI:** 10.1186/s12872-023-03171-5

**Published:** 2023-03-29

**Authors:** Nan Jin, Mengjie Yu, Xiaoyue Du, Zhiguo Wu, Changlin Zhai, Haihua Pan, Jinping Gu, Baogang Xie

**Affiliations:** 1 College of Pharmaceutical Sciences, Zhejiang University of Technology, Zhejiang, China; 2 Key laboratory of medical electronics and digital health of Zhejiang Province, Medical College of Jiaxing University, Jiaxing University, Jiaxing, China; 3 The Second Affiliated Hospital of Nanchang University, Nanchang University, Nanchang, China; 4 Department of Cardiovascular Diseases, Institute of Atherosclerosis, the Affiliated hospital of Jiaxing University, Jiaxing, China

**Keywords:** Children with pulmonary arterial hypertension associated with congenital heart disease (PAH-CHD), Metabolomics, Nuclear magnetic resonance, UPLC-MS/MS, Biomarkers

## Abstract

**Background:**

Pulmonary arterial hypertension is a common complication in patients with congenital heart disease. In the absence of early diagnosis and treatment, pediatric patients with PAH has a poor survival rate. Here, we explore serum biomarkers for distinguishing children with pulmonary arterial hypertension associated with congenital heart disease (PAH-CHD) from CHD.

**Methods:**

Samples were analyzed by nuclear magnetic resonance spectroscopy-based metabolomics and 22 metabolites were further quantified by ultra-high-performance liquid chromatography–tandem mass spectroscopy.

**Results:**

Serum levels of betaine, choline, S-Adenosyl methionine (SAM), acetylcholine, xanthosine, guanosine, inosine and guanine were significantly altered between CHD and PAH-CHD. Logistic regression analysis showed that combination of serum SAM, guanine and N-terminal pro-brain natriuretic peptide (NT-proBNP), yielded the predictive accuracy of 157 cases was 92.70% with area under the curve of the receiver operating characteristic curve value of 0.9455.

**Conclusion:**

We demonstrated that a panel of serum SAM, guanine and NT-proBNP is potential serum biomarkers for screening PAH-CHD from CHD.

**Supplementary Information:**

The online version contains supplementary material available at 10.1186/s12872-023-03171-5.

## Introduction

Congenital heart disease (CHD) is a common cardiovascular disease in pediatrics with a complex etiology associated with multiple factors. If CHD is not treated in a timely manner, the decrease in cardiac output, the increase in cardiac load, and the increase in pulmonary blood flow lead to an increase in mean pulmonary arterial pressure (MPAP), resulting in pulmonary arterial hypertension (PAH) and the loss of surgical opportunities in advanced irreversible PAH [1, 2]. Therefore, it’s critical to get a diagnosis and treatment as soon as possible in order to lower mortality rate and enhance quality of life for PAH-CHD patients. Right cardiac catheterization is the gold standard for the diagnosis of PAH, but it is an invasive interventional technique [3]. Thoracic echocardiography is a non-invasive method for the diagnosis of PAH, but this method is limited by the detection window of the trachea, and the display of the extracardiac structures is not clear [4, 5]. Biomarkers can be detected from blood, urine, feces and other body fluids, and the detection is convenient, low cost and non-invasive, which provides a new strategy for early diagnosis and treatment in the field of CHD-PAH. In recent years, studies have shown that brain natriuretic peptide (BNP), N-terminal pro- brain natriuretic peptide (NT-proBNP), asymmetric dimethylarginine (ADMA), and vascular endothelial growth factor (VEGF) may be potential diagnostic biomarkers for PAH-CHD, but the specificity of these biomarkers is still controversial [6, 7]. Therefore, it is of great clinical significance to screen suitable non- invasive biomarkers for early diagnosis of PAH-CHD from CHD in infants and young children.

With the development of high throughput analytical technology, omics-based analyses of DNA, RNA, proteins and metabolites are increasingly being applied to screen the diagnostic biomarkers [8]. As the final products of gene expression and protein alteration, metabolites not only reflect the changes in some functions of the body, but also reflect the overall metabolic changes of the body [9, 10]. Some biomarkers for the diagnosis and prognosis of cardiovascular diseases have been screened and identified by metabolomics [11–13]. Our previous study showed that the combination of four metabolites, namely, betaine, taurine, glutamine, and phenylalanine, can be used as potential serum diagnostic biomarkers in the children with CHD from the healthy controls by nontargeted proton nuclear magnetic resonance spectroscopy (^1^HNMR)-based and targeted ultra-high-performance liquid chromatography–tandem mass spectroscopy (UPLC–MS/MS)-based metabolomics [14]. However, these technologies have not been employed to screen and identify biomarkers of PAH-CHD in children.

**Fig. 1:**
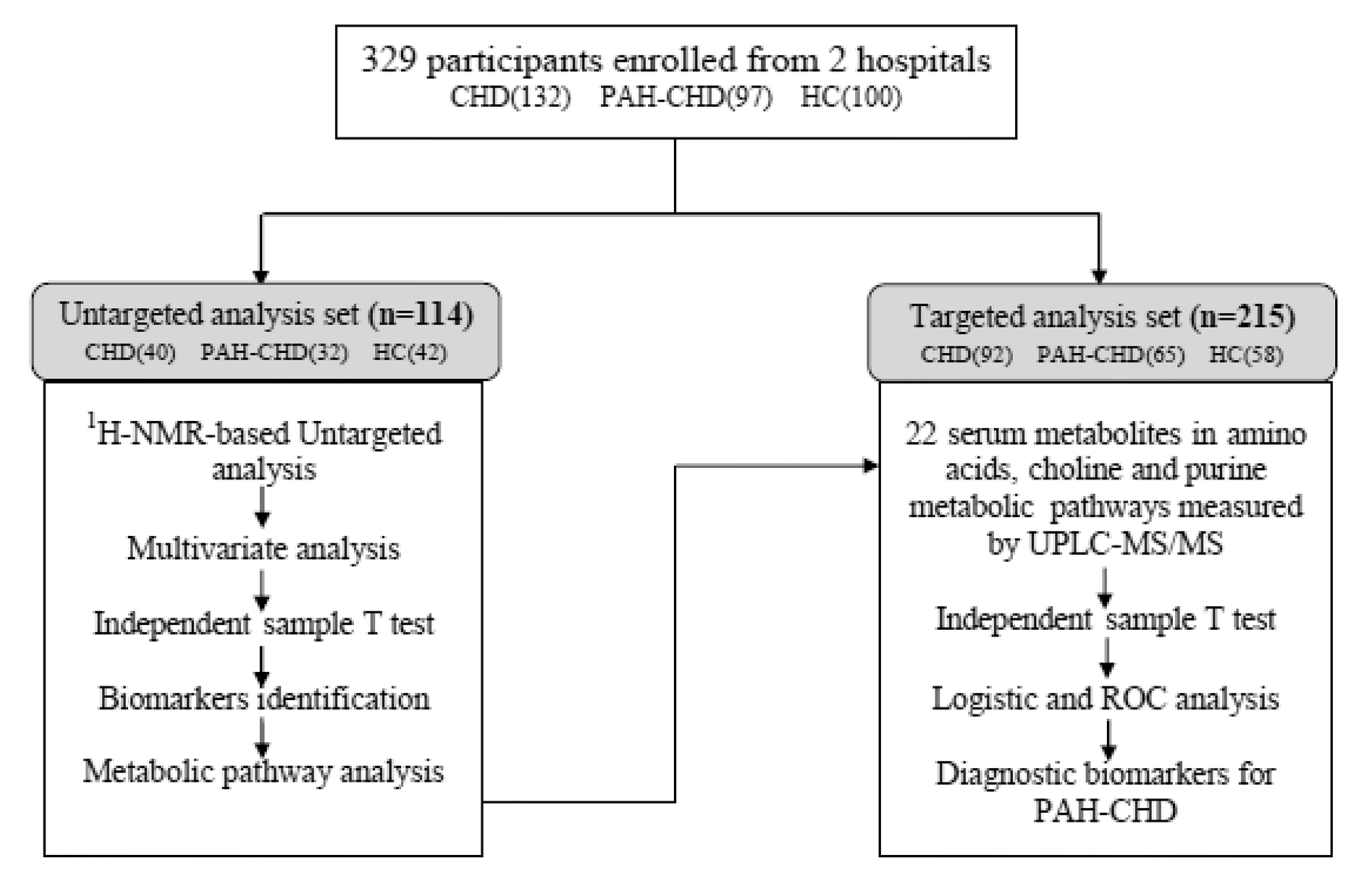
Experimental flowchart for screening the potential serum diagnostic biomarkers of PAH-CHD from CHD.

In this study, the serum of CHD, PAH-CHD and the healthy control (HC) children were investigated. Firstly, the ^1^HNMR based metabolomics were used to screen and identify the differential metabolites of CHD and PAH-CHD. Then, 22 serum metabolites were further quantified by UPLC-MS/MS. The quantitative data and clinical biochemical index were screened by binary logistic regression analysis and diagnostic efficacy of PAH-CHD and CHD by the metabolites combination was evaluated by ROC. The experimental flowchart of this study is shown in Fig. 1.

## Materials and methods

### Reagents and chemicals

Acetonitrile and methanol of LC-MS grade was purchased from Thermo Fisher Scientific (Shanghai, China). Phosphate buffer salts (NaH_2_PO_4_ and K_2_HPO_4_), D_2_O were purchased from Tianjin Weiyi Chemical Technology Co., Ltd (Tianjin, China). The following compounds were obtained from Sigma-Aldrich: ammonium formate and formic acid of LC-MS grade, 3-(Trimethylsilyl) propionic-2,2,3,3-d4 acid sodium salt(TSP), betaine, chloride, taurine, glutamine, glutamate, S-adenosvlmeIhionine (SAM), valine, leucine, methionine, tyrosine, xanthine, xanthosine, uric acid, guanosine, adenine, hypoxanthine, inosine, guanine of analytical grade (> 99%).

### Sample collection

Serum samples were collected from Jiangxi Children’s Hospital and the Second Affiliated Hospital of Nanchang University. All venous blood was collected in the morning of the second day after admission with fasting status before corrective surgery. The serum samples were kept under 4 °C temperature before stored at -80 °C within 6 h after serum isolation [15]. A total of 329 subjects were enrolled in this study, including 132 in group of CHD, 97 in group of PAH-CHD and 100 in group of HC. Written informed consent for participation was obtained and the study was reviewed and approved by The Ethics Committee of Jiangxi Children’s Hospital (No.Jxsetyy-yxky-20,200,101) in accordance with the Declaration of Helsinki.

### Sample preparation for ^1^HNMR analysis

The serum samples were thawed at room temperature and 200.0 µL of it was added to 800.0 µL methanol. After mixing, the samples were centrifugated at 12,000 g for 5 min. We took the 800.0 µL supernatant and dried at SpeedVac system (Hersey Instrument Co., Ltd, China) at the temperature of 45 ℃. We added 450.0 µL distilled water, 50.0 µL Phosphoric acid buffer solution(pH = 7.4)and 50.0 µL 5.0 mmol/L TSP in D_2_O solution to the residues. After mixing, samples were centrifuged at 12,000 g for 5 min, and 500.0 µL of supernatant was removed into the 5 mm NMR tube. The samples were stored at 4 ℃ before analysis.

The ^1^HNMR data were collected by Bruker AvanceII-600 MHz spectrometer (Germany) at 298 K temperature with Noesy pulse sequence according to previous report [16]. The water peak was inhibited by the presaturation method, and the 1D proton spectra were obtained from 64 scans over a spectral width of 14 ppm. Phase correction and baseline correction of all ^1^HNMR spectra were performed using Topspin 2.1 (Bruker, Biospin, GmbH, Rheinstetten, Germany), and the TSP chemical shift was calibrated to 0.00 ppm. In order to eliminate the influence of water peak and residual methanol peak, 4.6–5.4 ppm and 3.36-3.37ppm region data were deleted.

### Multivariate statistical analysis of ^1^HNMR data

The data of each spectral was normalized to the total spectral intensity over the entire spectrum. We imported the normalized data into SIMCA-P (version 14.0, Umetrics, Umea, Sweden), and completing multivariate statistical analysis such as principal component analysis (PCA) and orthogonal partial least squares discriminant analysis (OPLS-DA) [17]by using unit variance (UV) scaling preprocessing. The variables that cause the sample classification are obtained by the value of variable importance projection (VIP) [18]. The metabolites with VIP > 1.0 were identified by comparing the chemical shifts and coupling modes of the metabolites in ^1^HNMR spectra with the parameters in the literature and publicly accessible databases (http://www.bmrb.wisc.edu, http://www.hmdb.ca) and some standards were purchased for further identification of metabolites according to the method described in previous paper [19].

### Quantitative analysis of metabolites for classification of CHD and PAH-CHD by UPLC-MS/MS

The serum samples were thawed at room temperature, and 50.0 µL serum was added to 200. 0µL methanol, 25.0 µL 2-Aminoheptanedioic acid as internal standard (IS) dissolved in 80% methanol. The solution was further centrifuged at 14,000 g for 10 min, and 200.0 µL supernatant was put in the automatic sample bottle.

UPLC-MS/MS was performed by the ACQUITY UPLC system coupled with Xevo G2-XS QTof (Waters, USA). Chromatographic condition: ACOQUITY UPLC® BEH HILIC chromatographic separation column (2.1 × 100 mm, 1.7 μm; Waters, USA), 10.0 mmol/L ammonium formate + 0.01% formic acid aqueous solution (A) and acetonitrile (B), flow rate of 0.25 mL/min, injection volume of 3.0µL. The separation time was 5 min, and the following gradient elution was used: 0- 1.5 min, 20% A; 1.5-3.5 min, 20-80% A; 3.5-5 min 80% A. The temperature of the chromatographic column was set to 40℃, and the temperature of the automatic injector was set to 8℃. Mass spectrum condition: The metabolites were quantitatively analyzed by MS/MS under multiple reaction monitoring mode (MRM) with the positive electrospray ionization. The electrospray capillary voltage was set to 3.0 kV, and nitrogen was used as a dry gas for solvent evaporation, with a flow rate of 50 L/h. The temperature of the ion source was 100℃, and the Masslynx4.1 (Waters, USA) was used to control the instrument and data procession.

### Statistical analysis

Quantitative data were analyzed using IBM SPSS software package (22.0, IBM Corp). The K-S test was employed to determine the normality of distribution of the data. Normally distributed data were presented by mean and their standard deviation (SD). Abnormally distributed data were described using median and interquartile range. The ^1^HNMR peaks identified were integrated and the values of the peaks in CHD and PAH-CHD groups were compared with independent sample T test. Kruskal–Wallis test was used by comparing abnormally distributed quantitative data. Receiver operating characteristics (ROC) curve was employed to assess the predictive value of metabolite to predict PAH-CHD.

### Screening of optimal diagnostic marker combinations by binary logistic regression analysis

UPLC-MS/MS quantitative data and clinical biochemical data were integrated in a dataset. The binary logistic regression analysis was carried out with these variables. The model adopted the maximum likelihood estimation forward method to carry out stepwise regression analysis. The standard of the selected variables was p < 0.05. The criterion for eliminating the variables was p > 0.10. The optimal metabolite combination of the highest diagnostic efficiency was screened, and the diagnostic performance of the model was evaluated using ROC.

## Results

### General information of patients

According to the Sixth Pulmonary Hypertension World Symposium 2018, PAH is determined by the mean pulmonary artery pressure above 20 at rest, the group was divided based on measurement of pulmonary pressure in cardiac catheterization. 329 children including 132 cases in the CHD group, 97 in the PAH-CHD group and 100 in the HC group were enrolled in this study. The inclusion criteria are children with CHD including ventricular septal defect, atrial septal defect, Patent Ductus Arteriosus, and patent foramen ovale were enrolled. The patients with Tetralogy of Fallot were excluded. The exclusion criteria are the patients with chronic respiratory disease, acute heart failure, pulmonary venous hypertension, chronic liver or renal disease. The clinical information regarding patients is provided in Table 1. As shown in Table 1, some biochemical indicators such as urea nitrogen (BUN) and NT-proBNP were significantly altered when comparing PAH-CHD with CHD group.

**Table 1:** Baseline characteristics of overall participants

	CHD	PAH-CHD	HC	P-value CHD vs. PAH-CHD
Number (male/female)	132 (63/69)	97 (47/50)	100(48/52)	-
Age (years)	3.35 ± 2.05	2.83 ± 3.03	3.38 ± 3.10	0.1261
Weight(kg)	14.2 ± 5.0	13.4 ± 8.50	Not available	0.4467
Height (cm)	94.3 ± 14.3	80.9 ± 14.3	Not available	0.7692
WBC (10^9^/L)	9.71 ± 0.27	10.58 ± 0.44	10.32 ± 0.47	0.0973
LYMPH% (%)	55.56 ± 1.42	56.08 ± 56.08	56.69 ± 1.64	0.8190
LYMPH(10^9^/L)	5.37 ± 0.19	5.88 ± 0.31	5.94 ± 0.35	0.1646
LDH(U/L)	333.68 ± 5.85	353.58 ± 13.52	201.12 ± 15.32	0.1805
CK(U/L)	134.05 ± 5.80	115.49 ± 10.01	125.68 ± 8.90	0.1116
Mb(µg /L)	15.02 ± 0.68	14.53 ± 1.05	16.18 ± 2.36	0.6869
BUN(mmol/L)	4.45 ± 0.14	3.44 ± 0.17	3.79 ± 0.17	< 0.0001
CR(µmol/L)	25.49 ± 0.64	24.59 ± 0.90	25.25 ± 0.72	0.4037
UA(µmol/L)	254.25 ± 6.92	259.11 ± 12.82	257.64 ± 8.91	0.7396
CRP(mg/L)	1.71 ± 0.40	0.84 ± 0.22	1.3 ± 0.64	0.0573
SAA(mg/L)	20.59 ± 5.57	17.10 ± 4.90	6.87 ± 2.38	0.6565
NT-proBNP (pg/mL)	247.97 ± 17.72	1787.07 ± 202.68	Not available	< 0.0001

Note: WBC: White Blood Cell; LYMPH%: the percentage of lymphocytes; LYMPH: the count of lymphocytes; LDH: Lactate Dehydrogenase; CK: Creatine Kinase; Mb: Myoglobin; BUN: Urea Nitrogen; CR: Creatinine; UA: Uric Acid; CRP: C-reactive Protein; SAA: Serum Amyloid A; NT-proBNP: N terminal pro B type natriuretic peptide

### PCA and OPLS-DA analysis of ^1^HNMR data

**Fig. 2:**
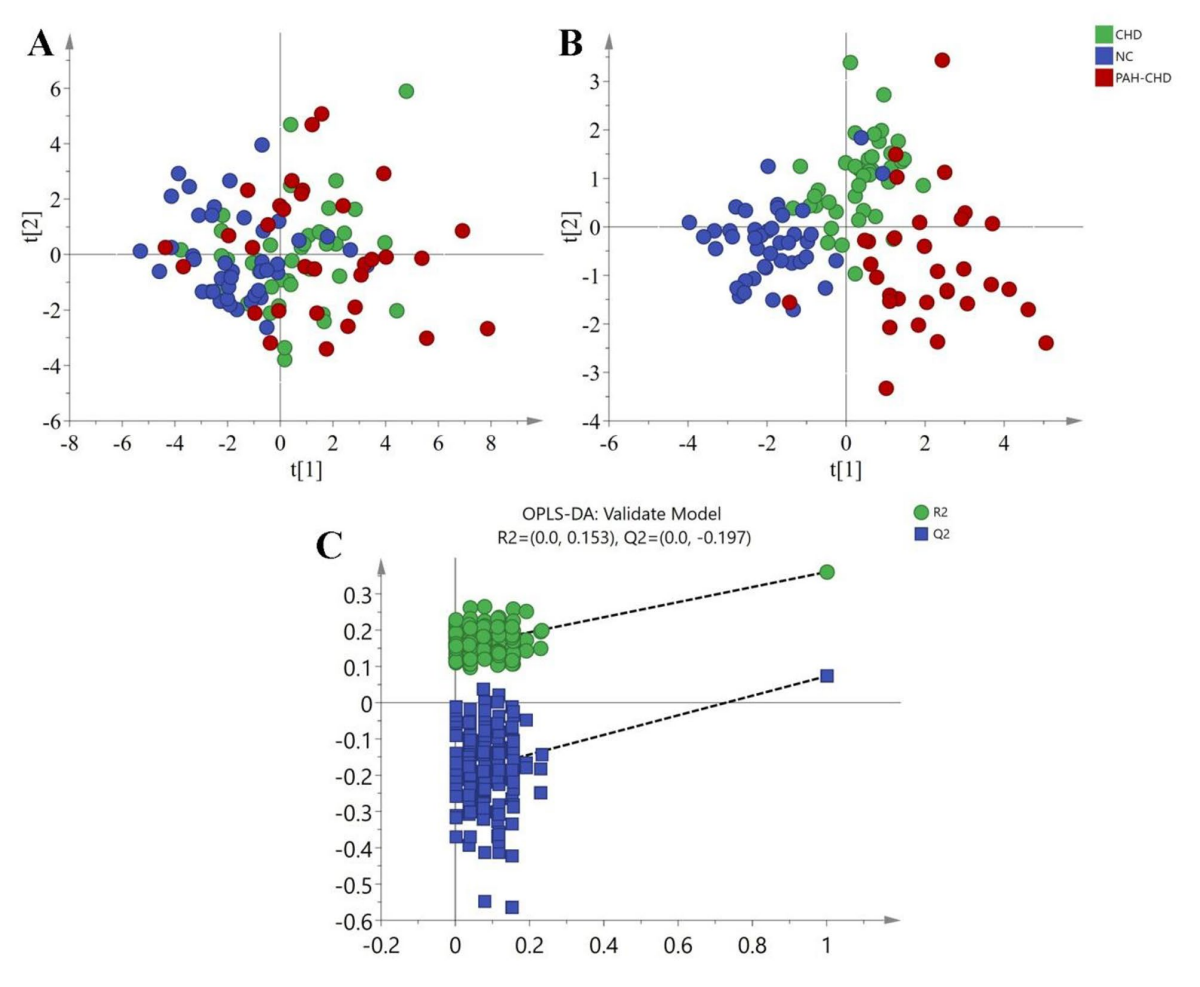
Result of PCA, OPLS-DA generated by data from ^1^HNMR spectra and permutation test plots (200 permutations) of the OPLS-DA model. (A) PCA scores plot; (B) OPLS-DA scores plot; (C) Permutation test plots (200 permutations) of the OPLS-DA model

The normalized data of ^1^HNMR were imported into SIMCA-P software for multivariate analysis. The PCA and OPLS-DA scores plot were shown in Fig. 2A and B. The quality of OPLS-DA model is usually evaluated by R^2^Y (cum) and Q^2^ (cum) two parameters. R^2^Y (cum) represents the explanatory power of the model, and Q^2^ (cum) represents the predictive power of the model. In general, when the values of R^2^Y (cum) and Q^2^ (cum) are more than 0.5, the model is better [20]. In this study, the three parameters of the OPLS-DA were (R^2^X = 0.407, R^2^Y = 0.54, Q^2^ = 0.315), which showed that the model was feasible. At the same time, we tested the reproducibility of the model and determined whether the model was overfitting by seven-fold cross validation and permutation test (200 times). The intercept value of the R^2^ and Q^2^ regression lines and the axis were used as the criterion to measure whether the model was over fitting. When the intercept of Q^2^ is negative value, the model is effective [21]. Figure 2 C showed that R^2^ = 0.153, Q2=-0.197, indicating that the model was effective. Our results indicated that OPLS- DA analysis could be used to detect the metabolic differences between the two groups of CHD and PAH-CHD.

### Metabolites contributing to the classification of CHD from PAH-CHD

**Fig. 3:**
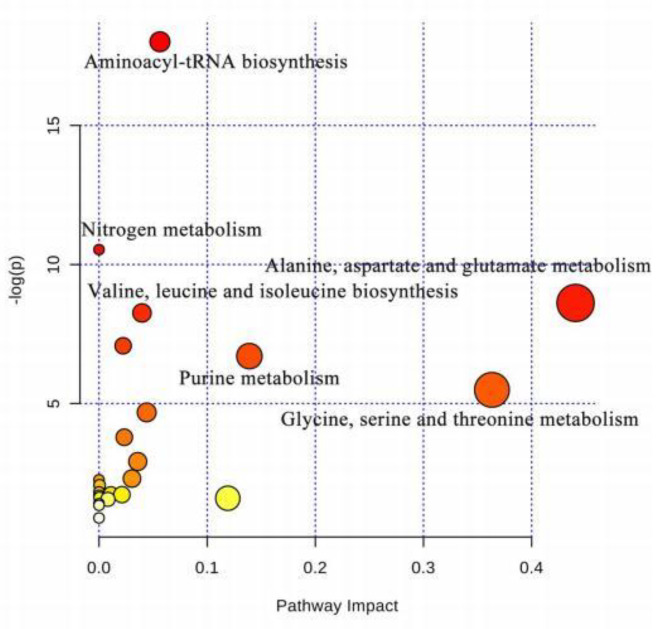
Significantly altered metabolic pathways between CHD and PAH-CHD patients All matched pathways were shown according to p values from the pathway enrichment analysis (y-axis) and pathway impact values from pathway topology analysis (x-axis) [23], with the most impacted pathways colored in red

The metabolites with variable importance (VIP) values greater than l.0 based on OPLS-DA model were supposed to the potential biomarkers for contributing to the classification of CHD from PAH-CHD [16, 22]. In this study, 12 metabolites of VIP > 1.0 were identified, as shown in Table 2 and Supplementary Figure s1. After the independent sample T test, the levels of 8 metabolites including leucine, valine, glutamate, glutamine, betaine, taurine, phenylalanine and xanthine were observed to be significantly changed (P < 0.05) in the PAH-CHD group. The biological pathways involved in the metabolism of these metabolites and their biological roles were determined by enrichment analysis using Metaboanalyst [23]. Results showed that 5 metabolic pathways including aminoacyl-tRNA biosynthesis, alanine, aspartate and glutamate metabolism, valine, leucine and isoleucine biosynthesis, purine metabolism, glycine, serine and threonine metabolism were altered in PAH-CHD group comparing with the CHD group (Fig. 3).

**Table 2:** Information of metabolites for classification of CHD and PAH-CHD by ^1^HNMR data

	Metabolites	δ^1^H	Integral interval	Relative levels in serum(mean ± SE)			VIP	P-value CHD vs PAH-CHD
				CHD (n = 40)	PAH-CHD (n = 32)	HC (n = 42)		
1	Leucine	0.95(t)	0.95–0.98	0.0093 ± 0.0007	0.0070 ± 0.0005	0.0103 ± 0.0006	1.1610	0.0066
2	Valine	1.02(d)	1.005–1.025	0.0732 ± 0.0042	0.0518 ± 0.0048	0.0901 ± 0.0035	1.3950	0.0012
3	Isoleucine	1.03(d)	1.03–1.055	0.0232 ± 0.0014	0.0270 ± 0.0020	0.0325 ± 0.0012	1.1237	0.1235
4	Alanine	1.48(d)	1.47–1.51	0.0014 ± 0.0001	0.0013 ± 0.0001	0.0018 ± 0.0001	1.1310	0.5082
5	Glutamate	2.34(m)	2.33–2.375	0.0351 ± 0.0012	0.0297 ± 0.0020	0.0293 ± 0.0010	1.1389	0.0261
6	Glutamine	2.44(m)	2.438–2.48	0.0762 ± 0.0025	0.0645 ± 0.0044	0.0662 ± 0.0022	1.0394	0.0242
7	Choline	3.19(s)	3.2-3.215	0.0081 ± 0.0004	0.0088 ± 0.0005	0.0058 ± 0.0003	1.3203	0.2839
8	Betaine	3.26(s)	3.261–3.278	0.0220 ± 0.0015	0.0559 ± 0.0052	0.0197 ± 0.0009	1.6773	< 0.0001
9	Taurine	3.42(t)	3.405–3.44	0.0156 ± 0.0007	0.0125 ± 0.0010	0.0135 ± 0.0007	1.3562	0.0088
10	Phenylalanine	7.42(m)	7.41–7.44	0.0557 ± 0.0039	0.0710 ± 0.0061	0.0835 ± 0.0030	1.4425	0.0381
11	Xanthine	7.80(s)	7.72–7.77	0.0117 ± 0.0008	0.0172 ± 0.0010	0.0100 ± 0.0004	1.1233	0.0001
12	Hypoxanthine	8.20(d)	8.19–8.22	0.3153 ± 0.0061	0.3040 ± 0.0125	0.3148 ± 0.0111	1.1354	0.4204

### Quantitative analysis of metabolites by UPLC-MS/MS

**Fig. 4:**
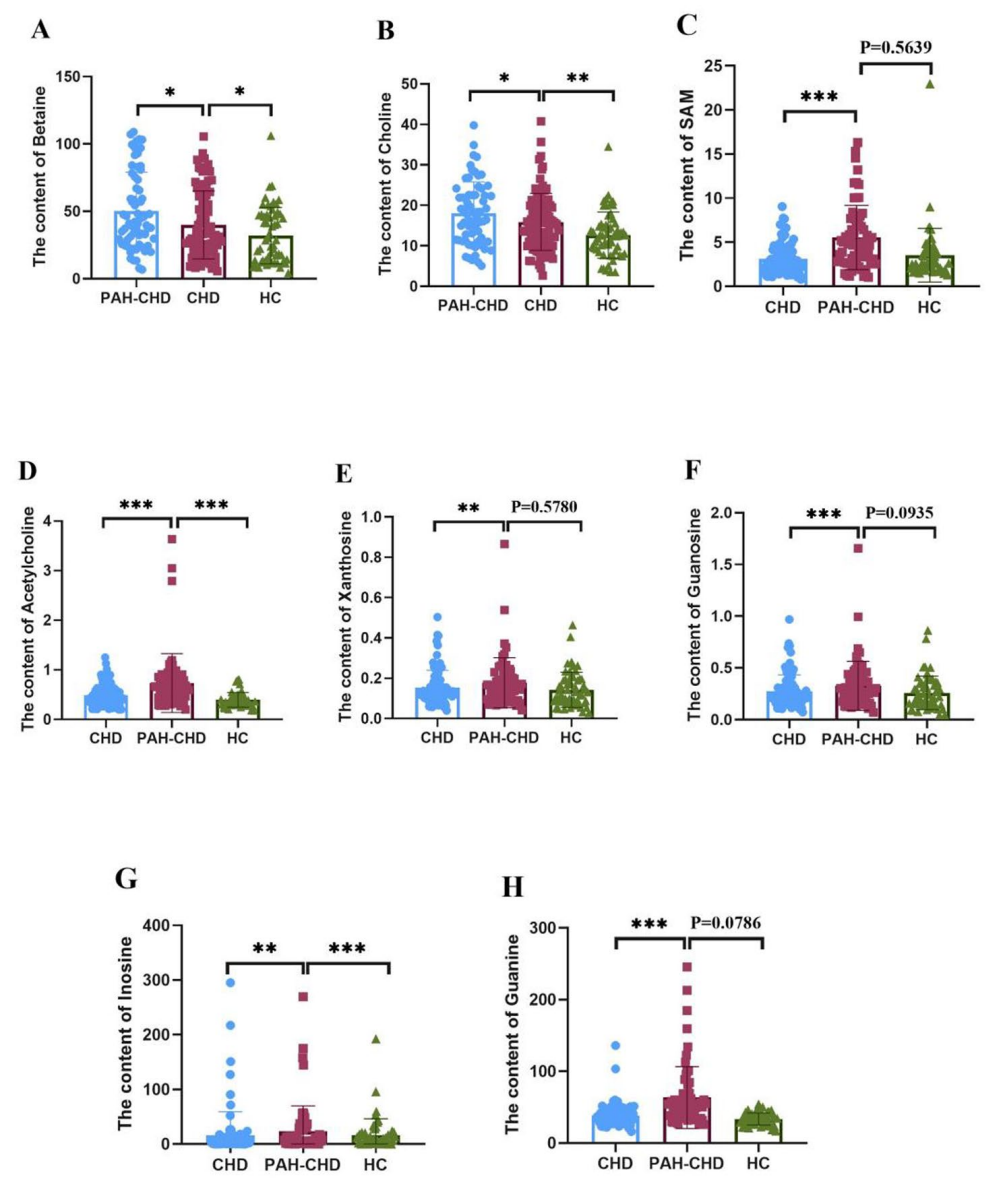
Significantly altered serum metabolites between PAH-CHD and CHD patients

Serum levels of 10 amino acids, 4 metabolite in choline metabolism pathway including choline, betaine, acety-choline, SAM, and 8 metabolites in purine metabolism pathway were further determined by established UPLC-MS/MS method with hydrophilic chromatography column in 92 cases of CHD, 65 PAH-CHD and 58 HC cases. The analysis method parameters were shown in the supplementary table s1 and table s2. As shown in Fig. 4 and supplementary table s3, compared with the CHD group, 8 metabolites including betaine, choline, S-Adenosyl methionine (SAM), acetylcholine, xanthosine, guanosine, inosine and guanine were significantly altered in PAH-CHD patients comparing with CHD (independent sample T test, P < 0.05).

### Binary logistic regression analysis for screening the best combination of diagnostic markers

The 8 metabolites which significantly altered in PAH-CHD, together with urea nitrogen (BUN) and N terminal pro B type natriuretic peptide (NT-proBNP) as a total of 10 variables were employed for binary logistic regression analysis. After the stepwise regression analysis, a total of 3 variables, SAM, guanine and NT-proBNP, were selected for the established logistic model. The model equation was Logit (p) = -5.394 + 0.057 × 1 + 0.020 × 2 + 0.008 × 3. The serum levels of SAM, guanine and NT-proBNP of the patients were X1, X2 and X3, respectively.

The values of the area under the curve (AUROC) of ROC curve were used to evaluate the diagnostic efficiency. The greater the AUC value, the more reliable the diagnostic effect is [24, 25]. As shown in Table 3, the AUROC value of each variable was obtained, we can see that most of metabolites were not good for diagnosis of CHD from PAH- CHD (AUROC < 0.77) except NT-proBNP (AUROC = 0.8812). The Logistic equation established with 3 variables of SAM, guanine and NT-proBNP showed 92.70% accuracy for the average prediction of the above 92 CHD and 65 PAH-CHD serum samples, and 0.9455 for AUROC values. The sensitivity and specificity were 0.8333 and 0.9873 respectively.

**Table 3:** The results of ROC analysis using the quantified data

	Metabolite	AUC	95% Confidence interval	Sensitivity	Specificity	Cut-off value
1	Betaine	0.6754	0.5421–0.8087	0.9259	0.3250	20.0995
2	Choline	0.5498	0.3899–0.7096	0.2963	0.8500	18.9784
3	SAM	0.7314	0.5934–0.8695	0.4074	0.9250	4.6542
4	Acetylcholine	0.6155	0.4630–0.7679	0.3333	0.8750	0.6423
5	Xanthosine	0.5826	0.4308–0.7344	0.4815	0.9250	0.1493
6	Guanosine	0.5923	0.4407–0.7438	0.5185	0.9000	0.2495
7	Inosine	0.6966	0.5551–0.8382	0.4815	0.8000	4.4907
8	Guanine	0.7594	0.6364–0.8824	0.5185	0.8750	42.9541
9	BUN	0.7356	0.5750–0.8963	1.0000	0.0345	1.5800
10	NT-proBNP	0.8812	0.8088–0.9535	0.8640	0.8480	492.2000

## Discussion

Firstly, we screened and identified the differential metabolites in 40 cases of CHD, 32 cases of PAH-CHD and 42 HC subjects by ^1^HNMR based metabolomic method. We found that the major metabolic pathways affected were amino acid metabolism, choline metabolism and purine metabolism. However, due to the limited sensitivity of ^1^HNMR, it is difficult for the identification and absolute quantitative analysis. Therefore, we established an UPLC-MS/MS method to quantify serum levels of 22 metabolites in 92 CHD patients, 65 PAH-CHD patients and 58 HC subjects. Our results showed that compared with the CHD group, serum contents of 8 metabolites, such as betaine, choline, SAM, acetylcholine, xanthosine, guanosine, inosine and guanine, were significantly altered(P < 0.05) in PAH-CHD group.

Zeneng Wang et al. reported that the increased levels of blood choline and betaine were associated with increased risk of heart disease [26, 27]. Our results showed that the contents of betaine, choline and acetylcholine increased in the CHD group as compared with the HC group. A metabolomic study showed that elevation of blood serotonin, taurine, creatine, sarcosine, and 2-oxobutanoate, and decrease of vanillylmandelic acid, 3,4-dihydroxymandelate, 15-keto-prostaglandin F2α, fructose 6-phosphate, l-glutamine, dehydroascorbate, hydroxypyruvate, threonine, l-cystine, and 1-aminocyclopropane-1-carboxylate in CHD-PAH group compared with the CHD group. Nevertheless, absolute quatificaion of these metabolites were not obtained in this study [28]. Here, we confirmed the increased contents of serum betaine, choline, acetylcholine and SAM in the PAH-CHD group by UPLC-MS/MS, which was similar to that reported in literature [29]. Our results indicated that during the process of CHD to PAH-CHD, the content of betaine, choline, acetylcholine and SAM increased continuously, suggesting that these 4 metabolites might be associated with the occurrence and development of CHD disease.

In addition, A Edlund et al. found that in patients with ischemic heart disease, metabolites in purine metabolism were largely released in the myocardium during myocardial ischemia, suggesting that ischemic heart disease might be associated with purine metabolism [30]. Recent study showed that purine metabolism were significantly disturbed in the PAH associated with the congenital Left-to-Right shunt (PAH-LTRS) cohort [31]. Our experimental results showed that the purine metabolites in the CHD group and the PAH-CHD group were significantly changed from those in the HC group. Compared with the CHD group, the contents of xanthosine, guanosine, inosine and guanine in the PAH-CHD group increased significantly (P < 0.05). Wei Sheng et al. reported a significant reduction in methylation levels in children with tetralogy of Fallot (TOF) and suggested that low methylation levels might increase the risk of TOF in children [32]. Some studies showed that betaine, choline, acetylcholine, SAM and xanthosine, guanosine, inosine and guanine were the metabolites related to the carbon metabolism, which are the common methyl donor for the methylation reaction [33–35]. Therefore, we speculated that methylation might be associated with the development of CHD. The accumulation of betaine, choline, acetylcholine, SAM, xanthosine, guanosine, inosine and guanine in children with PAH-CHD might be caused by the low methylation reaction of PAH-CHD children. In summary, the changes in metabolites in PAH-CHD and whether these alteration are related to the development of PAH-CHD are rarely reported, and we will further investigate the mechanism.

PAH is a common complication in late stage of CHD which has high morbidity and mortality, poor prognosis. Therefore, it is important to screen novel non-interventional biomarkers for diagnosis of PAH-CHD. George Giannakoulas et al. reviewed 26 studies related to PAH-CHD systematically, and found that compared with healthy controls, PAH-CHD patients had higher B type natriuretic peptide (BNP) and NT-proBNP, and they suggested that BNP might be simple and effective markers for the prognosis and timing of treatment intervention of PAH-CHD [6]. David M. et al. found that circulating endothelial cells could be a valuable tool to define therapeutic strategies in PAH-CHD patients. In addition, studies showed that sensitive cardiac troponins, connective tissue growth factor and growth differentiation factor-15 might be the diagnostic marker of PAH-CHD [36–38]. Compared with the difficulty and high cost of quantifying specific proteins and peptides, it is much easier to quantify serum small molecular metabolites.

Literatures reported that NT-ProBNP may be a potential biomarker of pulmonary hypertension [6, 39]. Consistent with the literature, we found that the NT-proBNP content in the PAH-CHD group was significantly increased (about 7 times, Table 1) compared with the CHD group. After binary logistic regression analysis, combinations of SAM, guanine and NT-proBNP were selected for the best diagnostic efficiency. Our results showed that the average prediction accuracy of 92 cases of CHD and 65 cases of PAH-CHD serum samples was 92.70%, the AUROC was 0.9455, and the sensitivity and specificity were 0.8333 and 0.9873 respectively. Therefore, serum SAM, guanine and NT-proBNP are expected to be potential biomarker combination for the differential diagnosis of PAH-CHD. However, a study on large number of clinical samples, metabolite flux and biomarker stability for serum collection should be conducted to further validate the results of this study.

## Conclusion

Screening novel serum biomarkers is of great clinical significance in improving the diagnosis of PAH-CHD. ^1^HNMR based metabolomics showed that the metabolic pathways of nitrogen, amino acids and purines were significantly altered in serum of PAH-CHD patients. Therefore, 22 metabolites were further quantified by UPLC-MS/MS. After binary logistic regression analysis, a biomarker panel consisting of SAM, guanine and NT-proBNP showed the best diagnostic efficiency. The average prediction accuracy of 92 cases of CHD and 65 PAH-CHD serum samples was 92.70% with AUROC of 0.9455. We therefore believe this 3-marker panel has the potential to be used in clinical practice for the early diagnosis and screening of PAH-CHD.

## Electronic supplementary material

Below is the link to the electronic supplementary material.

**Table s1:** The regression equation, limits of detection (LOD) and quantitation (LOQ).
**Table s2:** MRM quantitative parameters of metabolites quantified by UPLC-MS/MS.
**Table s3:** The content of serum metabolites quantified by UPLC-MS/MS.
**Figure s1:** VIP-coded loadings plot. The color scales (VIP values) show variable importance in the OPLS-DA projection generated by the serum 1H NMR data. 1, Leucine; 2, Valine; 3, Isoleucine; 4, Alanine; 5, Glutamate; 6, Glutamine; 7, Choline; 8, Betaine; 9, Taurine; 10, Phenylalanine; 11, Xanthine; 12, Hypoxanthine.

## Data Availability

The study data presented may be made available from the corresponding author upon reasonable request.
