# Supplementary material for: Identification of potential serum biomarkers for congenital heart disease children with pulmonary arterial hypertension by metabonomics
Source: BMC Cardiovasc Disord. 2023 Mar 29;23:167. doi: 10.1186/s12872-023-03171-5 (PMC10061882; doi:10.1186/s12872-023-03171-5)
Supplement: Supplementary file 1 — Table s1: The regression equation, limits of detection (LOD) and quantitation (LOQ). Table s2: MRM quantitative parameters of metabolites quantified by UPLC-MS/MS. Table s3: The content of serum metabolites quantified by UPLC-MS/MS. Figure s1: VIP-coded loadings plot. The color scales (VIP values) show variable importance in the OPLS-DA projection generated by the serum 1H NMR data. 1, Leucine; 2, Valine; 3, Isoleucine; 4, Alanine; 5, Glutamate; 6, Glutamine; 7, Choline; 8, Betaine; 9, Taurine; 10, Phenylalanine; 11, Xanthine; 12, Hypoxanthine. [file 12872_2023_3171_MOESM1_ESM.docx]

|  | Metabolites | Regression equation | r^2^ | Linearity(ng/mL） | LOD(ng/mL) | LOQ(ng/mL) |
| --- | --- | --- | --- | --- | --- | --- |
| 1 | Betaine | y=2.4417x+0.3708 | 0.9917 | 22.19-2840.91 | 1.55 | 12.50 |
| 2 | Choline | y=1.1044x+0.0368 | 0.9983 | 23.59-2930.51 | 2.19 | 19.00 |
| 3 | SAM | y=1.1117x-0.0210 | 0.9978 | 13.80-887.70 | 1.47 | 14.50 |
| 4 | Acetylcholine | y=32.093x+0.2822 | 0.9991 | 1.38-355.00 | 0.35 | 4.00 |
| 5 | Taurine | y=0.5803x+0.5696 | 0.9986 | 355.11-22727.20 | 11.10 | 120.00 |
| 6 | Valine | y=15.814x-1.6990 | 0.9905 | 208.01-1664.06 | 21.60 | 190.00 |
| 7 | Methionine | y=0.0182x-0.0279 | 0.9986 | 123.56-15326.24 | 10.30 | 110.00 |
| 8 | Glutamine | y=1.6004x+0.1047 | 0.9999 | 0.87-7102.27 | 0.03 | 0.30 |
| 9 | Glutamate | y=0.611x+0.1822 | 0.9975 | 221.95-28409.09 | 1.73 | 16.50 |
| 10 | Tyrosine | y=1.0711x-0.1695 | 0.9983 | 321.72-20589.77 | 12.33 | 130.50 |
| 11 | Phenylalanine | y=19.44x-0.5315 | 0.9978 | 146.65-1877.16 | 5.60 | 60.20 |
| 12 | Alanine | y=0.3972x-0.1705 | 0.9961 | 506.19-10123.86 | 2.23 | 25.00 |
| 13 | Leucine | y=0.7375x+0.4134 | 0.9946 | 232.92-14906.82 | 23.20 | 220.00 |
| 14 | Isoleucine | y=3.0865x-0.0602 | 0.9963 | 232.92-14906.82 | 12.65 | 130.00 |
| 15 | xanthine | y=0.0049x+0.291 | 0.9940 | 18.20-2560.00 | 0.20 | 0.60 |
| 16 | Xanthosine | y=0.0177x+0.012 | 0.9984 | 0.40-280.00 | 0.16 | 0.60 |
| 17 | Uric acid | y=0.000004x+0.001 | 0.9997 | 5.00-38200.00 | 0.15 | 0.55 |
| 18 | Guanosine | y=0.0175x+0.004 | 0.9901 | 0.30-20.50 | 0.07 | 0.28 |
| 19 | Adenine | y=0.0392x+0.077 | 0.9947 | 0.15-5.50 | 0.01 | 0.28 |
| 20 | Hypoxanthine | y=0.0045x+2.674 | 0.9968 | 100.00-2500.00 | 0.19 | 0.03 |
| 21 | Inosine | y=0.0007x+0.117 | 0.9935 | 0.01-2.20 | 0.01 | 0.60 |
| 22 | Guanine | y=0.0012x+0.002 | 0.9935 | 0.20-45.00 | 0.08 | 0.03 |

**Supplementary** Table s1: The regression equation, limits of detection (LOD) and quantitation (LOQ) .

**Supplementary** Table s2: MRM quantitative parameters of metabolites quantified by UPLC-MS/MS

|  | Metabolites | Ion transition(m/z) | Retention time(min) | CE Ramp | Cone Voltage |
| --- | --- | --- | --- | --- | --- |
| 1 | Betaine | 118.14→59.07 | 3.15 | 15 | 40 |
| 2 | Choline | 104.04→60.26 | 3.51 | 15 | 40 |
| 3 | SAM | 399.33→298.15 | 2.44 | 20 | 40 |
| 4 | Acetylcholine | 146.10→87.01 | 4.06 | 20 | 40 |
| 5 | Taurine | 126.09→108.01 | 1.58 | 15 | 40 |
| 6 | Valine | 118.06→103.05 | 2.24 | 20 | 40 |
| 7 | Methionine | 150.0→56.05 | 2.46 | 10 | 40 |
| 8 | Glutamine | 147.0→84.04 | 3.40 | 20 | 40 |
| 9 | Glutamate | 148.0→84.04 | 3.41 | 15 | 40 |
| 10 | Tyrosine | 182.09→165.05 | 2.32 | 15 | 40 |
| 11 | Phenylalanine | 166.08→120.08 | 2.26 | 15 | 40 |
| 12 | Alanine | 90.04→65.04 | 1.10 | 15 | 40 |
| 13 | Leucine | 132.0→91.05 | 1.04 | 15 | 40 |
| 14 | Isoleucine | 132.0→91.05 | 2.34 | 15 | 40 |
| 15 | Xanthine | 153.05→110.05 | 1.39 | 15 | 40 |
| 16 | Xanthosine | 285.1→152.08 | 1.44 | 20 | 40 |
| 17 | Uric acid | 169.04→141.05 | 1.46 | 15 | 40 |
| 18 | Guanosine | 284.1→152.07 | 1.46 | 20 | 40 |
| 19 | Adenine | 136.07→119.05 | 1.42 | 20 | 40 |
| 20 | Hypoxanthine | 137.07→110.05 | 1.43 | 20 | 40 |
| 21 | Inosine | 269.07→136.07 | 1.50 | 20 | 40 |
| 22 | Guanine | 152.07→135.05 | 1.02 | 20 | 40 |

**Supplementary** Table s3: The content of serum metabolites quantified by UPLC-MS/MS.

|  | Metabolites  (μg/ML) | CHD  (n = 92) | PAH-CHD  (n = 65) | HC  (n=58) | P-value  CHD vs PAH-CHD | P-value  CHD vs HC |
| --- | --- | --- | --- | --- | --- | --- |
| 1 | Betaine | 39.92±2.63 | 50.34±3.56 | 30.65±2.46 | 0.0173 | 0.0112 |
| 2 | Choline | 15.41±0.66 | 17.74±0.91 | 12.27±0.66 | 0.0351 | 0.0016 |
| 3 | SAM | 2.94±0.15 | 4.93±0.36 | 3.08±0.19 | <0.0001 | 0.5639 |
| 4 | Acetylcholine | 0.46±0.02 | 0.62±0.03 | 0.36±0.01 | <0.0001 | <0.0001 |
| 5 | Taurine | 45.13±1.81 | 40.85±2.41 | 28.27±1.78 | 0.1515 | <0.0001 |
| 6 | Valine | 8.25±0.40 | 7.33±0.43 | 7.56±0.45 | 0.1262 | 0.2691 |
| 7 | Methionine | 106.57±5.93 | 106.37±6.66 | 95.97±7.56 | 0.9828 | 0.2695 |
| 8 | Glutamine | 75.48±1.91 | 79.98±2.44 | 59.00±1.99 | 0.1454 | <0.0001 |
| 9 | Glutamate | 88.75±2.50 | 96.53±3.38 | 69.14±2.57 | 0.0606 | <0.0001 |
| 10 | Tyrosine | 6.43±0.25 | 6.47±0.36 | 6.88±0.31 | 0.9236 | 0.2564 |
| 11 | Phenylalanine | 8.84±0.46 | 8.82±0.57 | 10.15±0.56 | 0.9761 | 0.0763 |
| 12 | Alanine | 10.47±0.47 | 9.73±0.66 | 11.61±0.62 | 0.3426 | 0.1407 |
| 13 | Leucine | 17.86±0.93 | 17.06±0.77 | 15.23±0.92 | 0.5046 | 0.0555 |
| 14 | Isoleucine | 20.55±0.84 | 19.95±1.21 | 22.15±1.10 | 0.6717 | 0.2460 |
| 15 | xanthine | 5.94±0.29 | 6.52±0.30 | 3.48±0.17 | 0.1877 | <0.0001 |
| 16 | Xanthosine | 0.13±0.01 | 0.16±0.01 | 0.13±0.01 | 0.0021 | 0.5780 |
| 17 | Uric acid | 2415.45±87.46 | 2404.86±114.43 | 2109.31±88.36 | 0.9404 | 0.0208 |
| 18 | Guanosine | 0.21±0.01 | 0.29±0.02 | 0.24±0.02 | <0.0001 | 0.0935 |
| 19 | Adenine | 5.34±0.29 | 4.69±0.32 | 4.25±0.40 | 0.1457 | 0.0258 |
| 20 | Hypoxanthine | 134.03±4.04 | 137.78±6.37 | 124.44±5.96 | 0.6205 | 0.1856 |
| 21 | Inosine | 4.09±0.49 | 8.41±1.44 | 10.44±1.62 | 0.0060 | 0.0004 |
| 22 | Guanine | 36.36±0.96 | 47.57±1.88 | 33.72±1.12 | <0.0001 | 0.0786 |


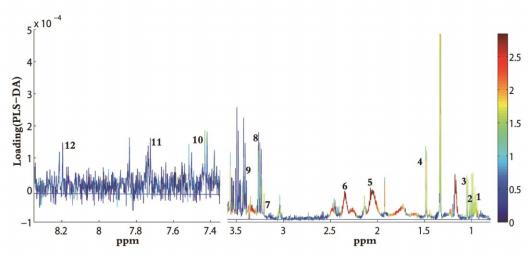


**Supplementary** Figure s1: VIP-coded loadings plot. The color scales (VIP values) show variable importance in the OPLS-DA projection generated by the serum 1H NMR data. 1, Leucine; 2, Valine; 3, Isoleucine; 4, Alanine; 5, Glutamate; 6, Glutamine; 7, Choline; 8, Betaine; 9, Taurine; 10, Phenylalanine; 11, Xanthine; 12, Hypoxanthine.
